# The Hospitals and the Coal-Porters' Strike

**Published:** 1914-01-31

**Authors:** 


### The Hospital

The Workers' Newspaper of Administrative Medicine and Institutional Life, Administration, National Insurance and Health.

No. 1440, Vol. LV. SATURDAY, JANUARY 31, 1914. PRICE ONE PENNY

### THE HOSPITALS AND THE COAL-PORTERS' STRIKE.

By the time these lines are in our readers' hands a settlement of the strike among the coalheavers will have been reached, according to current indications; and the danger of a coal famine in hospitals and other institutions for the sick appears for the moment to have been avoided. But a few days ago the situation was not so favourable, and many of the London hospitals were hard put to it for fuel wherewith to warm their wards, to sterilise their dressings, to boil their soiled linen, and so forth. It is no concern of institutional workers as such, or of The Hospital, how the strike arose, or whether the demands of the strikers were just. All that matters is that a strike for higher wages was declared, and that in consequence of it the work of the hospitals, both voluntary and rate-supported, was threatened with serious dislocation and impairment.

At the outset of the strike the union leaders seem to have decided that their followers should be allowed to load coal destined for the hospitals; and certain institutions actually received supplies in accordance with this policy. By last Saturday, however, a change of mind seemed to have occurred; and one hospital secretary, we hear, who telephoned to the strike leaders for permission to get in coals received abuse for his pains. According to a statement which has appeared in the press account of an interview with one of the strike organisers, the alteration of policy was due to a violation of the terms of the truce in respect of hospitals. He said that supplies of coal removed from a depot for the ostensible use of a hospital were partly conveyed elsewhere; and that, in consequence, it was decided to withdraw the privileges at first conceded to institutions for the sick. How much truth there is in this account it is impossible to say, since no details were given or names mentioned whereby the institution could be identified. Failing such details, we are entitled to say that until they are furnished we decline to believe that the episode took place as alleged. If any such breach of pact occurred, we feel quite certain that it was no fault of the institution in question; and we doubt very strongly whether the incident narrated really took place.

In any case, would the occurrence of one such episode have justified the strike committees in reversing their policy towards the hospitals? We think not, even if the breach of agreement could be directly traced to the hospital authorities. It would, certainly, justify a very sharp and plain warning from the union leaders; but not, in our judgment, such a penalty as they saw fit to enforce. And what if one of the alternate surmises be correct—namely, that the responsibility was none of the hospitals', or that the act of bad faith never took place at all? In the former event, the strike leaders were guilty of a piece of spiteful bad temper: in the latter, of a grave moral offence against the sick.

We should prefer to believe that the former charge is more credible than the latter; but some of the language that was used suggests that they are not overmuch troubled by scruples. One of their number, referring to the plight of the hospitals, is said to have made light of the matter, because in war people always have to suffer! True enough, of course; but has this militant coal-heaver never heard of the Red Cross flag, and the protective neutrality accorded to it on the battle-field? His simile is surely particularly unfortunate, for it amounts to exactly the same thing as shelling the ambulance flag indiscriminately on the ground of one (alleged) misuse of it. It will need better arguments, more subtle dialectics, than these to convince ordinary folk of the soundness of the strike leaders' contentions; and if the dispute had been a prolonged one, we fancy that public opinion would have made itself felt upon this point. It is evident that the strike leader who voiced the sentiment quoted regarded the coalmen as fighting not against the coalowners, but against the community; in other words, as Syndicalists holding the Metropolis to ransom. If this type of man has captured the union leadership, then it is plain that hospitals need expect no mercy. But the fact that no opposition was offered to the medical students who volunteered for the work of coalheaving on behalf of their respective hospitals, leads us to suspect that no such social revolution has yet occurred; and that the advanced views expressed about war are not those of the most responsible men among the strikers and their leaders. If it were so; if the coal-porters had been really bent on stopping the work of the hospitals as well as of the rest of industrial London (and all for an extra penny per ton), there can be no doubt that it would have been they and their families and the labouring classes in general who would have felt the pinch most. What the artisan classes would do if all the hospitals were shut down for want of coal we shudder to think of. The situation would be so intolerable that it could not last long; that is one comfort. And if there are those who contemplate such a prospect with equanimity, they may be recommended to study the lesson of a French play, produced a year or two ago in Paris, wherein a Syndicalist leader succeeded in plunging into darkness a hospital, where, unknown to him, his own child was being operated on for some dangerous condition. Anything more gruesome, while yet strictly within the bounds of possibility, than the *motif* of this play can hardly be conceived.

But let us end upon a note of cheerfulness. We do not believe the stoppage of coal supplies to hospitals was part of a Syndicalist plot, but rather the result of deplorable strategy. We do not think it has helped the strikers the least bit in the world, and we rejoice that it did not seriously hinder the work of the hospitals for the relief of sickness and suffering.

